# “外泌体分离分析专辑”引言

**DOI:** 10.3724/SP.J.1123.2025.01023

**Published:** 2025-05-08

**Authors:** Lianghai HU, Xiaoqiang QIAO, Weiguo TAO

## 引言

外泌体是由细胞分泌的脂质双分子层包裹的纳米级囊泡颗粒,携带多种核酸、蛋白质、代谢物与脂质等成分,广泛存在于各种体液中,是细胞间通讯的重要介质。外泌体的分离是其下游分析和应用的前提,高纯度、高收率、高通量的分离方法将有助于发挥外泌体在临床诊疗中的应用潜力;外泌体颗粒及其组分的分析可为标志物发现及药物递送提供关键的科学依据。

要解锁外泌体蕴含的巨大潜力,高效精准的分离和分析技术是关键前提。近年来,科研人员在外泌体分离分析领域取得一系列重要进展,为了集中展示我国学者在外泌体研究领域的最新成果,展望未来研究方向,受《色谱》期刊委托,我们组织了“外泌体分离分析专辑”,邀请了国内高等院校和科研院所等相关领域专家学者为本刊撰稿。

本专辑由3篇视角、6篇专论与综述和5篇研究论文组成,涉及外泌体的分离富集(体积排阻色谱、亲和配体、核酸适配体、靶向多肽、微流控技术、分子印迹、抗体磁珠、密度梯度离心等),外泌体亚群与成分分析(蛋白质组学、代谢组学、脂质组学、单细胞与单颗粒分析等),外泌体在标志物筛选和药物递送中的应用等研究领域。我们衷心希望通过本专辑的出版,充分展示我国学者在外泌体研究领域的最新成果,推动学科交叉与合作研究,为生命健康领域的发展贡献力量。

衷心感谢各位作者及审稿专家的鼎力支持和奉献!

本专辑客座主编

吉林大学 胡良海 教授

河北大学 乔晓强 教授

普渡大学 陶纬国 教授

2025年1月15日

